# Machine learning determination of applied behavioral analysis treatment plan type

**DOI:** 10.1186/s40708-023-00186-8

**Published:** 2023-03-02

**Authors:** Jenish Maharjan, Anurag Garikipati, Frank A. Dinenno, Madalina Ciobanu, Gina Barnes, Ella Browning, Jenna DeCurzio, Qingqing Mao, Ritankar Das

**Affiliations:** Montera Inc. dba Forta, 548 Market St, San Francisco, CA PMB 89605 USA

**Keywords:** Machine learning, Artificial intelligence, Autism spectrum disorder, Applied behavioral analysis

## Abstract

**Background:**

*Applied behavioral analysis (ABA) is regarded as the gold standard treatment for autism spectrum disorder (ASD) and has the potential* to improve outcomes for patients with ASD. It can be delivered at different intensities, which are classified as comprehensive or focused treatment approaches. Comprehensive ABA targets multiple developmental domains and involves 20–40 h/week of treatment. Focused ABA targets individual behaviors and typically involves 10–20 h/week of treatment. Determining the appropriate treatment intensity involves patient assessment by trained therapists, however, the final determination is highly subjective and lacks a standardized approach. In our study, we examined the ability of a machine learning (ML) prediction model to classify which treatment intensity would be most suited individually for patients with ASD who are undergoing ABA treatment.

**Methods:**

Retrospective data from 359 patients diagnosed with ASD were analyzed and included in the training and testing of an ML model for predicting comprehensive or focused treatment for individuals undergoing ABA treatment. Data inputs included demographics, schooling, behavior, skills, and patient goals. A gradient-boosted tree ensemble method, XGBoost, was used to develop the prediction model, which was then compared against a standard of care comparator encompassing features specified by the Behavior Analyst Certification Board treatment guidelines. Prediction model performance was assessed via area under the receiver-operating characteristic curve (AUROC), sensitivity, specificity, positive predictive value (PPV), and negative predictive value (NPV).

**Results:**

The prediction model achieved excellent performance for classifying patients in the comprehensive versus focused treatment groups (AUROC: 0.895; 95% CI 0.811–0.962) and outperformed the standard of care comparator (AUROC 0.767; 95% CI 0.629–0.891). The prediction model also achieved sensitivity of 0.789, specificity of 0.808, PPV of 0.6, and NPV of 0.913. Out of 71 patients whose data were employed to test the prediction model, only 14 misclassifications occurred. A majority of misclassifications (*n* = 10) indicated comprehensive ABA treatment for patients that had focused ABA treatment as the ground truth, therefore still providing a therapeutic benefit. The three most important features contributing to the model’s predictions were bathing ability, age, and hours per week of past ABA treatment.

**Conclusion:**

This research demonstrates that the ML prediction model performs well to classify appropriate ABA treatment plan intensity using readily available patient data. This may aid with standardizing the process for determining appropriate ABA treatments, which can facilitate initiation of the most appropriate treatment intensity for patients with ASD and improve resource allocation.

**Supplementary Information:**

The online version contains supplementary material available at 10.1186/s40708-023-00186-8.

## Introduction

Autism spectrum disorder (ASD) is a complex, life-long neurodevelopmental disorder which expresses heterogeneously in afflicted patients and is characterized by deficits in social communication and social interaction, as well as the presence of restricted, repetitive patterns of behavior, interests, and activities ([1–3]). Approximately 1 in 100 children worldwide are diagnosed with ASD [4], and the Centers for Disease Control and Prevention (CDC) estimates that approximately 1 in 44 children 8 years of age in the United States (US) have been identified as having ASD [5]. If left untreated or if treatment is insufficient, children with ASD may not develop competent skills with regards to learning, speech, or social interactions [6, 7]. Further, adults with ASD who have not received appropriate treatment may have difficulty living independently, maintaining employment, developing and maintaining relationships, and are at greater risk for both physical and mental health issues [6].

Although many therapeutic approaches exist, applied behavioral analysis (ABA) treatment is considered by many as the gold standard for ASD treatment [8, 9, 10, 10, 11]. Effective ABA treatment relies upon an early ASD diagnosis and determination of appropriate ABA treatment intensity to improve prognosis, which can generally be defined as a better quality of life, ranging from significant gains in cognition, language, and adaptive behavior to more functional outcomes in later life [8, 2, 11]. Owing to the heterogeneity of ASD, an individualized treatment plan is a defining feature and integral component of ABA treatment. The type of ABA treatment plan is conventionally determined by a trained Board Certified Behavior Analyst (BCBA) via integrated assessment of information derived from detailed patient intake forms, patient and family goals, and functional analysis of the patient [8]. Presently, there are two types of recognized ABA treatment plans, as defined by the level of intensity and target domains. A focused ABA treatment plan is provided directly to the patient for a limited number of behavioral targets, typically ranging from 10–20 h per week [8, 11]. A comprehensive ABA treatment plan targets all developmental domains (cognitive, communicative, social, and emotional) impacted by the patient’s ASD and typically ranges in intensity from > 20–40 h per week [8, 11]. Determination of whether to pursue comprehensive versus focused ABA treatment for a given patient is intrinsically subjective and inconsistent [12]. Although the Behavior Analyst Certification Board (BACB) provides guidelines for this determination [8], there is no standardized approach. BCBAs must frequently conduct re-assessments to ensure the patient is still experiencing a positive clinical response from the selected type of treatment, which further compounds the subjective nature of determining the most beneficial type of ABA treatment for a given patient over time. Although it has been clearly documented that comprehensive ABA treatment plans significantly improve clinical outcomes in patients with ASD [9–11], data also indicate that similar clinical benefits can be achieved with focused ABA treatment plans for certain patients with an ASD diagnosis that require a lower level of support [10, 11]. This finding is consistent with the most recent guidelines set forth in the Diagnostic and Statistical Manual of Mental Disorders, Fifth Edition (DSM-5), which clearly establishes that different patients have different needs in terms of support depending on the “severity” of symptoms [1].

Although ABA treatment has been shown to improve clinical outcomes in patients with ASD, there are challenges and significant considerations associated with determining which type of ABA treatment plan intensity (focused or comprehensive) is most appropriate for the affected patient, as well as their family and the ASD community at-large. First, although several studies have demonstrated that outcomes (e.g., mastery of learning objectives) are linearly related to the number of treatment hours per week (i.e., “intensity” of treatment), there is considerable variability in treatment responses and, as such, many patients with ASD benefit significantly with less intense treatment [10, 11]. Second, there is a substantially greater demand for BCBAs and registered behavior technicians (RBTs) than practically available to treat all patients with ASD [13–18], and shortages of ASD support services are well documented. In the US, 53%-94% of states report shortages of various ASD support services [17]; shortages of trained ABA therapists are reported in 49 states based on the Analyst Certification Board caseload recommendations [18]. Shortages of appropriately trained ABA therapists have also been documented outside of the US, in countries where ABA therapy is commonly sought by families to treat ASD [19–22]. This can lead to substantial delays in access to care. Clearly identifying patients that may benefit from a focused ABA treatment plan, which requires fewer resources, may increase the availability of current professionals (e.g., BCBAs and RBTs) to treat more patients and ensure those patients who most need a comprehensive ABA treatment plan will receive the appropriate level of care. Third, there is a significant financial burden associated with ABA treatment plans, with the cost of a comprehensive ABA treatment plan being considerably greater than that of a focused ABA treatment plan (e.g., cost of a comprehensive ABA treatment plan of 30–40 h/week vs. cost of a focused ABA treatment plan of 10–20 h/week). Similarly, the time burden is much greater for a comprehensive compared with a focused ABA treatment plan. The collective effect of these latter stressors is not trivial, as caregivers of patients with ASD have been shown to exhibit higher rates of stress, depression, anxiety, and other mental health disorders than the caregivers of patients having other developmental delays and disabilities [23, 24].

Recently, a range of machine learning (ML) techniques and models have been developed to assist in the diagnosis of ASD and to better understand neurological and genetic biomarkers which might be related to the condition [25–33]. Advances in patient data availability through the widespread adoption of electronic health records (EHRs) and the use of publicly available de-identified data through databases which contain records including demographic information, clinical assessments, and medical history can also allow for predictions related to ABA treatment plans using software only applications, for example, by employing ML prediction models [34]. Given the complex and heterogeneous nature of ASD, and the individualization of ABA treatment plan required, ML can be a powerful tool to improve the standardization of ASD treatment plan type determination, and ultimately maximize clinical outcomes, particularly in children. A small pilot study by Kohli et al. examined the use of ML to identify goals that would be most beneficial to ASD patients undergoing ABA treatment, irrespective of whether the type of ABA treatment was focused or comprehensive [34]. Their prediction model achieved an area under the receiver-operator characteristic curve (AUROC) of 0.78–0.80 for identifying specific skills to target, demonstrating the potential value of integrating ML into ASD treatment planning [34]. ML classification of an ABA treatment plan as either focused or comprehensive could also be helpful for newly trained BCBAs and RBTs to improve decision-making regarding ABA treatment plan types, and could potentially offer a more standardized approach for determining the plan type. ML may be particularly useful as this specialty field continues to grow as a direct result of increased healthcare needs owing to a higher number of patients being clinically-diagnosed with ASD [14, 15, 4]. Therefore, the purpose of the present study was to develop an ML prediction model for ABA treatment plan type determination using readily available information solely from patient intake forms for patients who were diagnosed with ASD by a licensed professional.

## Methods

### Dataset information

All patients included in this retrospective study (*n* = 359 patients ranging from 1 to 50 years of age) were referred to Montera Inc. dba Forta for ABA treatment after being diagnosed with ASD according to DSM-5 criteria by a licensed professional. The retrospective data were obtained from patient intake forms for ABA treatment completed by the parents, caregivers, or patients themselves for the 359 individuals diagnosed with ASD and provided with ABA treatment by Montera Inc. dba Forta. No patient data were excluded from this retrospective study. The ABA patient intake form utilized for all 359 patients included questions regarding demographics, schooling, behavior, skills, and goals related to the clients. The methods described in this paper did not utilize any identifiable data for data analysis, feature engineering, training of the machine learning model, or testing/validation of the model. The human subjects research conducted herein has been determined to be exempt by Pearl Institutional Review Board per Food and Drug Administration 21 Code of Federal Regulations (CFR) 56.104 and 45CFR46.104(b)(4) (#22-MONT-101).

The full dataset encompassing 359 patient forms was divided into a training dataset and a hold-out test dataset in an 80:20 split (i.e., training dataset: 288 forms, hold-out test dataset: 71 forms). The data from the 288 forms in the training dataset were used for feature selection, as well as cross-validation and optimization of hyperparameters. The data from the 71 forms in the hold-out test dataset were never exposed to the model during the feature selection or during cross-validation and optimization of hyperparameters, and were used solely as a hold-out test dataset to evaluate the efficacy of the ML prediction model. In other words, the hold-out test dataset remained completely independent of the training process and was solely used to evaluate the performance of the ML prediction model. The ground truth for the type of treatment received was determined based on the average number of hours of ABA treatment prescribed by a BCBA and authorized by insurance for a patient to receive weekly. The prevalence of patients who received comprehensive ABA treatment in the full dataset was 27.6% (99 patients). The remaining patients from the full dataset received focused ABA treatment (260 patients).

The ABA patient intake form includes various information about the patient as listed in Additional file 1: Table S1, mostly in the form of simple yes/no or multiple choice questions. It should be noted that these questions and answers from the intake forms were used by the BCBAs to determine the treatment plan intensity (i.e., the number of hours of ABA treatment) for each patient, and these BCBA determinations were the ground truth for each patient. It should be further noted that the BCBAs employ their knowledge and expertise to analyze the questionnaire answers and deliver a recommendation of the number of hours of ABA treatment for each patient, which recommendation is intrinsically subjective. The BCBAs do not have an algorithm to follow in their interpretation of the questionnaire answers, they simply abide by the BACB guidelines and utilize personal experience in the field to inform their recommendation of how many hours of ABA treatment a given patient should receive. These same questions and answers (listed in Additional file 1: Table S1) were used as features for the model’s analysis, ensuring that the data used by both the model and the BCBAs to determine treatment plan type (i.e., focused vs. comprehensive) were identical. Owing to the nature of the patient intake form, the data collected from this questionnaire have numerous binary and categorical inputs, which increases the dimensionality of the data. Thus, the process of feature processing and feature selection becomes critical. Some of the techniques implemented in the data processing and feature selection process are described below.

### Data processing and feature selection

**Fig. 1:**
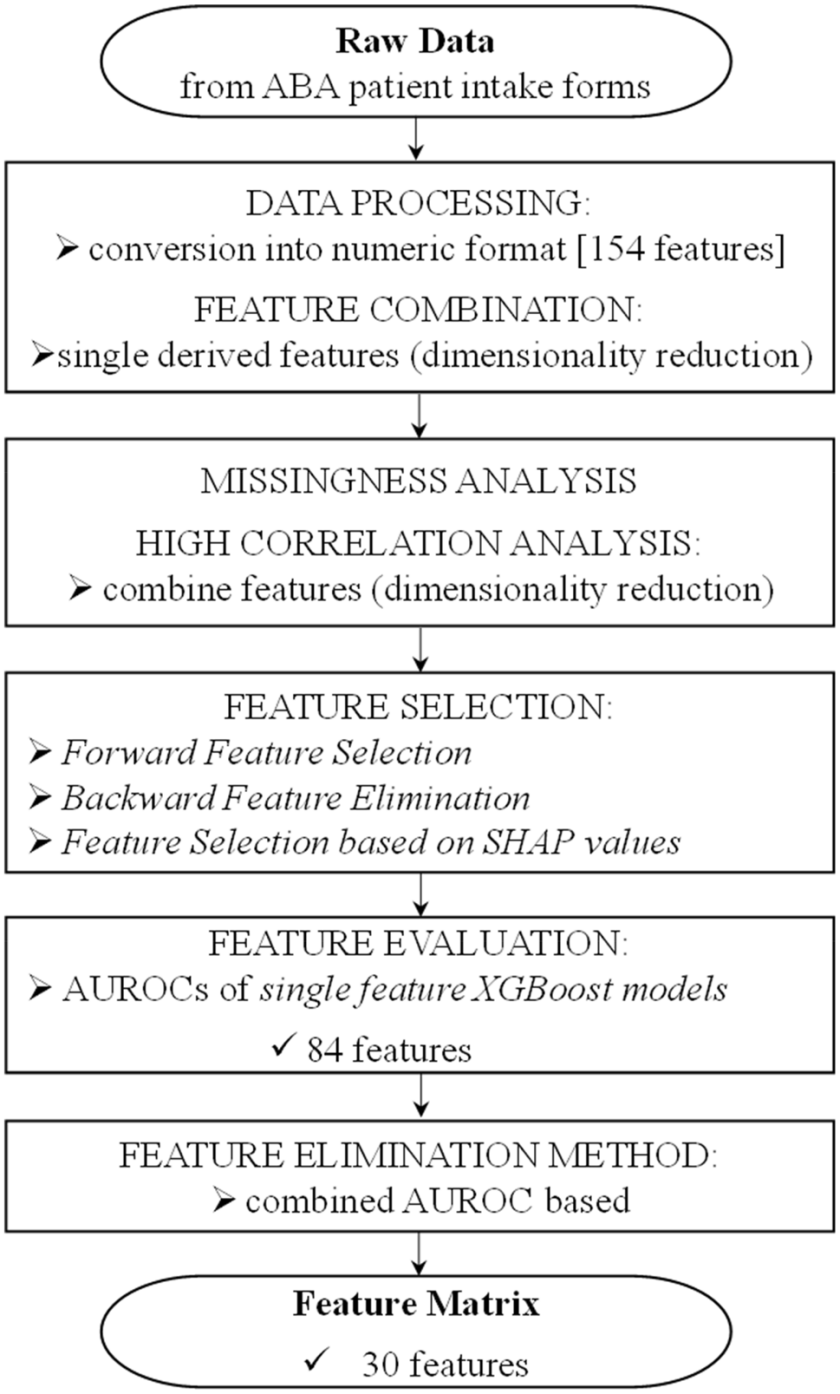
Data processing and feature selection flowchart. The raw data from the ABA intake forms were processed and subjected to rigorous feature selection to generate the feature matrix used to train and test the machine learning (ML) prediction model. *ABA* applied behavioral analysis; *SHAP* SHapley Additive exPlanations; *AUROC* area under the receiver-operator characteristic curve

As previously discussed, the features that were selected for the model to generate an analysis were identical to the intake categories that were used by the BCBAs to make a determination of focused versus comprehensive ABA treatment plans. The raw data collected from ABA patient intake forms included a variety of information about the patients, including their demographic information, medical history, records of the past treatment, schooling, parental medical history, behavioral assessment, skills assessment, and expected parent goals. The data from all 359 patients were processed and subjected to rigorous feature selection to generate the feature matrix used to train and test the ML prediction model. The feature matrix is the final input data matrix consisting of the individual patients as rows and their input features as columns. Figure 1 displays a flowchart outlining the data processing and feature selection applied to the raw data from the intake forms in order to generate the feature matrix used to train and test the ML prediction model.

Data processing encompassed converting all the data into numeric format. Most of the data in the ABA patient intake forms were in a textual form, either as categorical values or yes/no type questions. The categorical values were either one-hot encoded (e.g., *Sex* was one-hot encoded into two columns: *Male, Female*) or converted into an ordinal type value (e.g., *How severe is the child's aggressive behavior?*: Mild = 1, Moderate = 2, Severe = 3). After conversion to numeric format, some of the input features in the data were combined to create a single derived feature. For example, the feature “Aggression Score” was derived from three variables by multiplying their values: (i) “Does the child display aggression?” with possible values of Yes (1) and No (0); (ii) “How frequently does the child exhibit aggression?” with possible values of Less often than weekly (0), Weekly (1), Daily (2) and Hourly (3); and (iii) “How severe is the child's aggressive behavior?” with possible values of Mild (1), Moderate (2), Severe (3). A patient who exhibited moderate aggressive behavior on a daily basis would have a value of 4 (1 × 2 × 2, respectively) for “Aggression Score.” If a patient did not exhibit any aggressive behavior or exhibited aggressive behavior less often than weekly, the Aggression Score would be 0 (1 × 0 × 1, respectively). Combining such binary and numeric inputs into a single numeric input for a particular feature (e.g., aggression score) allowed capturing the relevant information while decreasing the number of inputs in order to maintain a suitable dimension of the feature matrix.

After processing the data, an analysis of missingness of input features and the correlation between input features was performed. While the chosen ML model (XGBoost) is able to handle missing data, a high rate of missingness in the data can lead the model to draw wrong conclusions [35], and thus, input features were checked for a high missing rate (> 50%). No features had a missing rate of over 50% and thus no features were eliminated due to missingness. Further, inputs that were highly correlated (correlation coefficient *r* > 0.85) with other features were eliminated. For example, input features ‘*Male’* and *‘Female,’* which both represent sex, were highly correlated and thus one of the features was eliminated and the other one retained as an input feature. Highly correlated features provide similar information to the model and thus, removing highly correlated features helps reduce the dimensionality of the data and also addresses the concerns of computational complexity without hampering the model’s performance. If not mitigated, high dimensionality can also lead to difficulties in the model’s ability to identify the features of most importance [36].

After removing features with a high rate of missingness and highly correlated features, various additional feature selection methods including *Forward Feature Selection, Backward Feature Elimination* and *Feature Selection based on SHapely Additive exPlanations (SHAP) values* were run on the remaining features to generate heuristics on the predictive capabilities of each feature [37, 38], essentially using the model to illuminate which features contribute the most to the model’s predictions. *Forward Feature Selection* evaluates features by incrementally adding features to the model, *Backward Feature Elimination* successively eliminates features from the model; and *Feature Selection based on SHAP values* evaluates features based on their importance for the model prediction. SHAP feature prediction (i.e., *Feature Selection based on SHAP values*) utilizes the SHAP values of a model trained on all of the original features to filter out the features showing the least importance. This approach allows for an earlier understanding of the features which contribute the most to the model’s predictive capabilities. As SHAP values provide an indication of the magnitude of a feature’s impact on the model, they can be used as a feature selection method during the feature engineering process. As described under the *Results* and *Discussion* sections below, subsequent to feature selection and model training, SHAP values can be employed in methods of evaluating the model, for example to further interpret the model following training and testing. It should be noted that the SHAP plots used in the method of interpreting the prediction model only include the final features utilized in the prediction model. It should be further noted that the SHAP values employed in the feature selection process evaluate all of the features in order to arrive to feature matrix, and thus the method of *Feature Selection based on SHAP values* and the *SHAP-based method of interpreting the prediction model* are different and distinct from each other. The three methods of feature selection (i.e., *Forward Feature Selection, Backward Feature Elimination* and *Feature Selection based on SHAP values*) can be used sequentially, in parallel, or a combination of sequentially and in parallel. However, we employed them in parallel (i.e., these feature selection methods were used independently of each other), and their results were evaluated for commonly emerging features. The features emerging independently from each other in each of the three methods of feature selection were further targeted for either elimination or retention based on their predictive capabilities.

We also trained single feature XGBoost models to predict whether a patient requires a comprehensive ABA treatment plan or a focused ABA treatment plan. In order to understand the discriminative quality of each of the features, the AUROC of each single feature XGBoost model was evaluated. Based on the results of the aforementioned data processing and feature selection methods including the evaluation based on the AUROCs of the single feature XGBoost models, we pruned the feature set down from 154 to 83 features, ensuring that at least one feature representing each of the various aspects of the patients, such as demographics, schooling information, parental medical history, behavioral assessments and goal related information, was preserved. The remaining set of 83 features was subjected to a feature elimination method based on a combined AUROC method as described below.

**Fig. 2:**
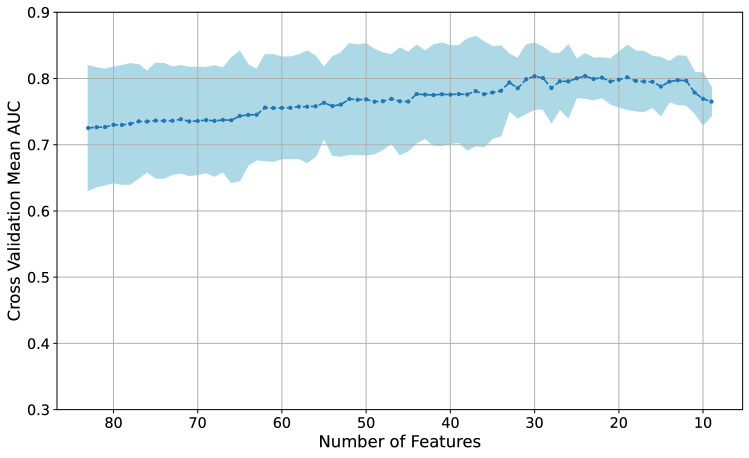
Cross-validation area under the receiver-operator characteristic curve (AUROC) vs. number of features. *AUROC* area under the receiver operator characteristic curve

Subsequent to the preliminary data processing and the feature selection methods described above, we employed the combined AUROC-based feature elimination method by building a baseline model using the remaining 83 features. This baseline model was an XGBoost model with low tree depth (maximum tree depth of 2) and 100 decision trees, where these particular values were chosen to prevent overfitting. We then trained additional XGBoost models using the same set of hyperparameters for each of these additional XGBoost models, where the training was performed iteratively by removing one feature at a time with replacement from the feature set (i.e., the set encompassing 83 features). These additional XGBoost models were trained using cross-validation, and the mean of cross-validation AUROC was used as the main performance metric. It should be noted that feature subsets were not reshuffled between folds. We observed that removing certain features improved the model performance more than the others. In order to examine the effect of feature removal, the single feature which led to the highest increase in the mean of cross-validation AUROC when removed was eliminated from the feature set to avoid data overfitting. We repeated this process of elimination on the remaining set of features until we observed a sustained decline in the mean of cross-validation AUROC as displayed in Fig. 2, which shows the variation in the mean of the cross-validation AUROC as features are eliminated in the combined AUROC-based feature elimination method. The combination of features that achieved the highest AUROC while capturing the various aspects of the patient’s data was selected as the set of features to train the final ML prediction model. By using the combined AUROC-based feature elimination method, we further pruned the feature set down from 83 to 30 features, which are listed in Table 1.

**Table 1:** List of input features used to train the machine learning prediction model. The table also shows the type or category (e.g., demographics, schooling information, parent’s medical history, past treatment and therapy, behavioral assessments, etc.) of the input features, and is a subset of all available inputs (shown in Additional file 1: Table S1). The input features were selected from the larger list of features by using the feature selection process described in the *Methods* section (and outlined in Fig. 1). Inputs in bold font were derived by combining multiple inputs within the category

ML prediction model input categories	ML prediction model input features
Demographics	Age
Schooling	Attends school?
	Grade
	Child received additional services as part of IEP/ARD
	Child has a school aide/support during school hours
Parent’s medical history	Mother or Father has history/presence of depression or manic-depression
	Mother or Father has history/presence of substance abuse or dependence
	Mother or Father has history/presence of anxiety disorders (OCD, phobias, etc.)
	Mother or Father has history/presence of attention-deficit hyperactivity disorder (ADHD)
Treatment/therapy	Amount of prior ABA treatment (hours of treatment per week)
	Amount of prior ABA treatment (years)
	History of Occupational Therapy: Has the patient ever received Occupational Therapy?
	History of Speech Therapy: Has the patient ever received Speech Therapy?
Behavioral assessment	Does the child destroy property?
	**Aggression Score: Level of the patient’s engagement in aggressive behavior derived from the frequency and severity of aggression**
	Does the child engage in stereotypy?
	**Stereotypy Score: Level of the patient’s engagement in Stereotypical repetitive behavior derived from the frequency and severity of stereotypy behavior**
Consequences for misbehavior	**Consequences Count—How many of the 'consequences for misbehavior' options were checked off**
Communication	Understanding—strangers can usually understand child
	Understanding—parent can usually understand child
Feeding and drinking habit	**Food Choice Score: Score indicating food choice behaviors**
Toileting and bathing skills	**Bathing Ability: Ability of patients to bathe themselves**
	**Toileting Independence Score: Score indicating independence in toileting skills**
Stim/RRB count	**Stim/RRB Count: Count of Stim/RRB options checked for a patient**
Expected parent goals	Expected Parent Goals—improve communication skills
	Expected Parent Goals—learn to eat healthier/more balanced diet
	Expected Parent Goals—learn to be more independent
	Expected Parent Goals—new ways to express frustration or when upset
Medical history	Medication for Sleep
	Medication for Allergies

*IEP/ARD* individual education program/admission, review and dismissal; *OCD* obsessive compulsive disorder; *ADHD* attention-deficit hyperactivity disorder; *Stim/RRB* self-stimulatory/restricted, repetitive behaviors

### Model training

The ML prediction model was trained using the feature matrix with the 30 input features chosen during the feature selection process described above for all 288 patients in the training dataset. The ML prediction model, an XGBoost-based model, is a gradient-boosted tree ensemble method of ML which combines the estimates of simpler, weaker models—in this case shallow decision trees—to make predictions for a chosen target [39]. The recent research has used gradient-boosted tree algorithms for acute and chronic prediction tasks with high accuracy. This includes sepsis prediction, long-term care fall prediction, non-alcoholic steatohepatitis or fibrosis, neurological decompensation, autoimmune disease, and more [40–46]. One of the benefits of using XGBoost is that it can implicitly handle a certain level of missingness in the data by accounting for missingness during the training process [47]. This is achieved by the model assigning the given feature with missing values to a default “node” on the model’s decision tree based on the best model performance after the feature has been assigned to a given node [47]. The ability to implicitly handle missing data is of particular importance when data are collected from individuals through a form vs. an automated collection, as individuals are more likely to submit incomplete data from which a determination of treatment plan type still needs to be performed. XGBoost has also been shown to perform better than other ML models on tabular data, which made it an appropriate choice for our dataset [48].

**Fig. 3:**
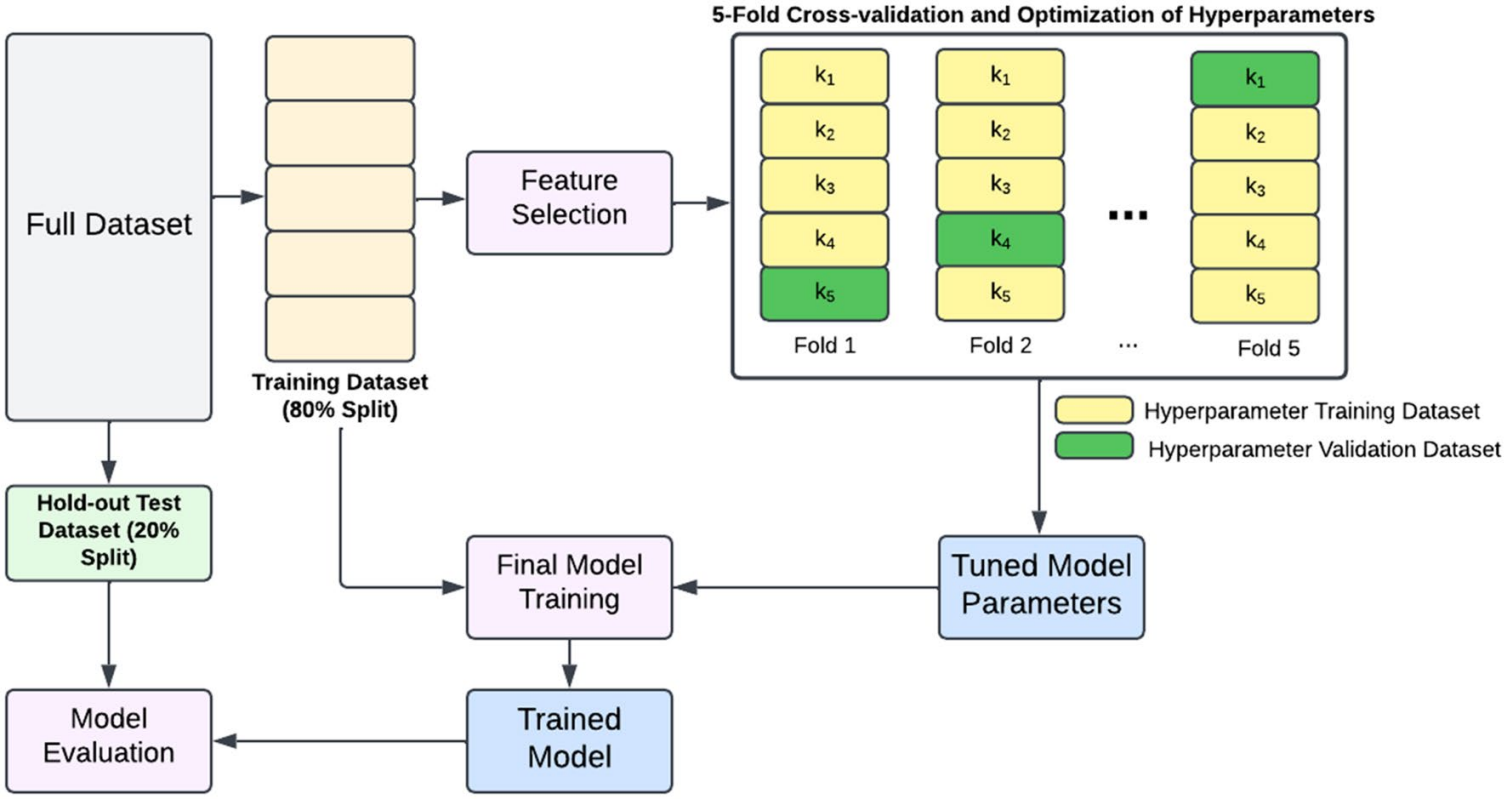
Model training and evaluation workflow. The training dataset was used for feature selection and optimization of hyperparameters using a fivefold cross-validation method. The tuned model hyperparameters were then used to train the machine learning prediction model with the full training dataset. The trained model was then evaluated on the hold-out test dataset

Before training the ML prediction model, hyperparameter optimization was performed using the training dataset, subsequent to the feature selection process and as shown in Fig. 3. Similar to the feature selection process, the hyperparameter optimization process also used the cross-validation method to tune the hyperparameters. Cross-validation is a resampling method that uses different portions of the data (i.e., data subsets) to test and validate a model on different iterations [49]. In this case, the 288 data points in the training dataset were divided into 5 random and equal subsets after which an XGBoost model was trained on 4 of them and validated on the remaining one. This was repeated using each of the 5 subsets as a validation set, and the subsets were not reshuffled between folds. The method of cross-validation allows for building a model more robust to variability in the data, i.e., a more generalizable model. Hyperparameters were optimized using a grid search method, from *Scikit-learn,* which takes each possible combination of hyperparameters and runs through cross-validation with each combination [50].

The three main hyperparameters tuned using the grid search method included *maximum tree depth, number of decision trees* and *scale positive weight*. The *maximum tree depth* hyperparameter determines the complexity of the weak learners—it limits the depth of the contributing decision trees, thereby controlling for the number of features which are part of the classification of each weak learner. Relatively lower range of values between 2 and 4 were selected for tuning *maximum tree depth* in order to develop a more conservative model, thus, limiting weak learners which overfit to the specific feature values of the training dataset. The *number of decision trees* determines the number of rounds of boosting (a method of combining the estimates of the weak learners by taking each weak learner sequentially and modeling it based on the error of the preceding weak learner). Higher values for the *number of decision trees* would increase the risk of model overfitting. Thus, the search grid for the *number of decision trees* was kept under 500. *Scale positive weight* is tuned to manage the class imbalance in the dataset. This hyperparameter represents the ratio of the positive to negative class samples utilized to build each of the weak learners in the model, allowing the model to sufficiently learn from the data of the class with a lower prevalence in the dataset. This hyperparameter’s search grid was set with values close to the ratio of the counts of two classes. Other hyperparameters including *learning rate* and regularization coefficients (*alpha, gamma*) were optimized as well. *Learning rate* determines how quickly the model adapts to the problem. A lower learning rate is preferred as lower learning rates lead to improved generalization error [51]. *Regularization* parameters are used to penalize the models as they become more complex in order to find sensible models that are both accurate and as simple as possible [52]. The final set of hyperparameters values were: *maximum tree depth* = *2, number of decision trees* = *100, scale positive weight* = *2.65, alpha* = *0.1, gamma* = *0.05,* and *learning rate* = *0.25.* Once the optimal hyperparameters were obtained, the ML prediction model was trained using the training dataset (i.e., 288 patients). The ML prediction model was then evaluated on the hold-out test dataset.

In addition to the cross-validation for the hyperparameter tuning, a separate k-fold cross validation was performed with 5 additional random train-test splits (5 folds) to further ensure the reliability of the results. This was performed after the hyperparameters had been optimized and was performed by creating 5 additional train-test splits and training a new model on each data fold. This additional model was evaluated for the same performance metrics as the primary model and used as a supplemental comparison.

### Comparison with other machine learning models

To gauge the performance of the XGBoost-based ML prediction model versus models built with other machine learning algorithms, a random forest model was trained. The random forest model required additional data processing (by comparison to the data processing done for the XGBoost-based ML prediction model) to successfully train the model. In contrast to XGBoost models, random forest models cannot handle null values implicitly, and thus any data points with null values either had to be removed from the dataset or replaced. For features with time ordinal data, such as hours of past ABA, as well as binary variables based on family medical history, null values were replaced with an impossible extreme value (i.e., a value so extreme it could not occur in reality). As random forests utilize the values of variables and do not infer a relationship within the variable values, using an extreme value does not affect the model’s performance. However, for null values in data representing behavior, such as bathing ability, rows with null values were removed from the random forest training set as excessive replacement of null values with extreme values can lead to erratic or inaccurate behavior [35]. After this imputation and filtering, the final size of the training set utilized for the random forest model was 212 patients with the same 30 features employed for training the XGBoost-based ML prediction model. The random forest model was trained on this 212 patients dataset and tested on a separate hold-out test consisting of 46 patients after data filtering. Further, as data are collected through the response of individuals on a form, missing data constitute a natural part of the data collection process and while XGBoost can implicitly handle this missing data, additional techniques are required to process missing data in other algorithms, such as the random forest.

### Comparison with standard of care

As the current standard of care for ABA treatment plan type determination is multifactorial and encompasses a high degree of subjective clinical judgment [8, 11, 12], no direct comparator exists against which to measure the ML prediction model performance. Consequently, we developed a “standard of care” comparator encompassing the features that are specified by the BACB in their treatment guidelines to contribute to the decision of a focused vs. a comprehensive ABA treatment plan [8]. The features selected from the ABA patient intake form for constructing the standard of care comparator (i.e., comparator features) encompass (per BACB guidelines) the types of behaviors exhibited by a patient, the number of behaviors exhibited by the patient, and the number of targets to be addressed for that particular patient. The standard of care comparator accounted for the following features as inputs into the comparator: age, restricted and repetitive behaviors, social and communication behaviors, listening skills, aggressive behaviors, and total number of goals to be addressed. The comparator features were utilized in combination by the standard of care comparator to determine which care plan should be recommended, as described below. It should be noted that the standard of care comparator is a mathematical proxy comparator that we have developed for the purpose of our research, and thus it is not a tool available to BCBAs. As described above in the *Dataset Information* sub-section, the BCBAs employ their knowledge and expertise to recommend a specific number of hours of ABA treatment for each patient, which recommendation is intrinsically subjective.

A linear regression function was constructed to enable combining the inputs to the standard of care comparator in order to generate a determination of a focused vs. a comprehensive ABA treatment plan. This linear regression function generated an output score which was a linear combination of the comparator features. The inputs for the linear regression function were obtained from the data processed to train and test the ML prediction model (i.e., the processed data of the training dataset prior to the feature selection process described above for the ML prediction model). The score generated by the linear regression function serves as a proxy, accounting for the BACB guidelines [8], for the manual assessment process that the BCBA follows in order to determine whether a patient should receive focused or comprehensive ABA treatment. Scores generated by the linear regression function were then compiled into a receiver-operating characteristic (ROC) curve, and an operating point was selected on the ROC curve to determine which scores of the comparator indicated a focused ABA treatment plan and which scores indicated a comprehensive ABA treatment plan. The operating point value is the cutoff determining which class (e.g., focused ABA treatment plan or comprehensive ABA treatment plan) to which a particular output belongs. The operating point for the linear regression function ROC curve was selected to meet the desired sensitivity of the ML prediction model. As the chosen operating point for the ML prediction model corresponded to a sensitivity of 0.789, the operating point for the linear regression function was also selected as the point on the ROC curve with similar sensitivity in order to allow for an appropriate comparison. As a measured sensitivity of 0.789 was not available with the random forest model, the nearest measured sensitivity of 0.75 was utilized to compare performance.

### Evaluation metrics

The AUROC was used as the primary metric to evaluate the performance of both the ML prediction model, the random forest model, and the linear regression function used as a proxy for the standard of care. Other metrics used to evaluate the performance of the ML prediction model, the random forest model, and for comparison with the standard of care were calculated as follows:

$$Sensitivity\, = \,\frac{{No.\,~of\,~patients~\,correctly\,~classified\,~by~\,the~\,model~\,~as~\,needing\,~comprehensive~\,treatment\,~plan}}{{No.~\,of~\,patients~\,who~\,received\,~comprehensive~\,treatment~\,plan~\,\left( {ground\,~truth} \right)}}$$

$$Specificity\, = \,\frac{No. \,of \,patients\, correctly \,classified \,by\, the \,model \,as\, needing\, focused \,treatment \,plan}{{No. \,of \,patients\, who\, received \,focused \,treatment\, plan \,\left( {ground \,truth} \right)}}$$

$$Positive\,Predictive\,Value\,(PPV)\, = \, \frac{{No. \,of\, patients \,correctly\, classified\, by \,the\, model \,as \,needing \,comprehensive \,treatment\, plan}}{{ No. \,of \,patients\, who\, were \,classified\, by\, the \,model\, as \,needing \,comprehensive\, treatment \,plan}}$$

$$Negative \,Predictive \,Value\, \left( {NPV} \right)\, = \,\frac{No. \,of\, patients\, correctly \,classified\, by\, the\, model\, as \,needing \,focused\, treatment \,plan}{{No. \,of\, patients\, who \,were\, classified\, by \,the\, model \,as \,needing\, focused\, treatment \,plan}}$$

Note: In the above equations, ‘the model’ refers to the ML prediction model, the random forest model, or the linear regression function for the comparator.

### Statistical analysis

The confidence intervals (CIs) for AUROC were calculated using a bootstrapping method. For the bootstrapping method, a subset of patients from the hold-out test dataset were randomly sampled and the AUROC was calculated using the data from those patients. This step was repeated 1000 times with replacement. From these 1000 bootstrapped AUROC values, the middle 95% range was selected to be the 95% CI for the AUROC. As the sample size of the hold-out test dataset was greater than 30, the CIs for other metrics were calculated using normal approximation [53].

## Results

### Subject population and characteristics

Subject demographics for patients in the training dataset separated by ABA treatment plan type (comprehensive or focused) are shown in Table 2. Overall, the average age was 6 years (range: 1–50 years), and as expected, there was a greater number of males compared with females (*P* < 0.05; [12]. There was a greater relative number of younger patients (< 5 years) with ASD in the comprehensive ABA treatment group than the focused ABA treatment group (53% vs. 26%). In contrast, there was a lower relative number of older patients (> 8 years) with ASD in the comprehensive ABA treatment group than the focused ABA treatment group (24% vs. 41%). Within the comorbidities present, there was a greater relative prevalence of anxiety/depression and attention-deficit hyperactivity disorder (ADHD) in the focused group. Subject demographics for patients in the hold-out test dataset separated by ABA treatment plan type (comprehensive or focused) are shown in Table 3, and the overall demographics were similar to those in the training dataset.

**Table 2:** Demographics table showing the breakdown of the patients in the training dataset based on the age, sex and (ASD severity). The table also shows the distribution of various comorbidities between the two types of ABA treatment plans

Demographics		Comprehensive (*N* = 80) (%)	Focused (*N* = 208) (%)
Age (years)	0–5	41 (51.2)	49 (23.6)
	5–8	19 (23.8)	71 (34.1)
	8-older	20 (25.0)	88 (42.3)
Sex	Male	61 (76.2)	155 (74.5)
	Female	17 (21.2)	53 (25.5)
	Unknown sex	2 (2.5)	0 (0.0)
ASD Severity	Mild	25 (31.2)	34 (16.3)
	Moderate	24 (30.0)	87 (41.8)
	Severe	30 (37.5)	66 (31.7)
	No severity Information	1 (1.2)	21 (10.1)
Comorbidities	Anxiety and depression	1 (1.2)	15 (7.2)
	ADHD	7 (8.8)	48 (23.1)
	Intellectual and language disorders	10 (12.5)	20 (9.6)
	Developmental delay	3 (3.8)	8 (3.8)

*ASD* autism spectrum disorder; *ABA* applied behavioral analysis; *ADHD* attention-deficit hyperactivity disorder

**Table 3:** Demographics table showing the breakdown of the patients in the hold-out test dataset based on the age, sex, and ASD severity. The table also shows the distribution of various comorbidities between the two types of ABA treatment plans

Demographics		Comprehensive (*N* = 19) (%)	Focused (*N* = 52) (%)
Age (years)	0–5	11 (57.9)	14 (26.9)
	5–8	5 (26.3)	14 (26.9)
	8-older	3 (15.8)	24 (46.2)
Sex	Male	15 (78.9)	42 (80.8)
	Female	4 (21.1)	10 (19.2)
	Unknown	0 (0.0)	0 (0.0)
ASD Severity	Mild	0 (0.0)	5 (9.6)
	Moderate	7 (36.8)	14 (26.9)
	Severe	9 (47.4)	24 (46.2)
	No severity info	3 (15.8)	9 (17.3)
Comorbidities	Anxiety and depression	0 (0.0)	3 (5.8)
	ADHD	2 (10.5)	12 (23.1)
	Intellectual and language disorders	1 (5.3)	5 (9.6)
	Developmental delay	1 (5.3)	2 (3.8)

*ASD* autism spectrum disorder; *ABA* applied behavioral analysis; *ADHD* attention-deficit hyperactivity disorder

### Feature selection and ML model optimization

The results from the combined AUROC-based feature elimination method are shown in Fig. 2. Starting from the initially pruned down feature set of 83 features, as features were eliminated one after another based on the combined AUROC-based elimination method described in the *Methods* section, a gradual increase in AUROC was observed with a maximal value being achieved with 24 feature inputs. The average cross-validation AUROC close to the maximal value attained during the process (~ 0.80) occurred for three sets of features—sets with 30, 27 and 24 features. Thus, in order to allow the model to make decisions based on as much information as possible, the set of 30 features was used as the final set of inputs (i.e., feature matrix).

### ML model performance

**Fig. 4:**
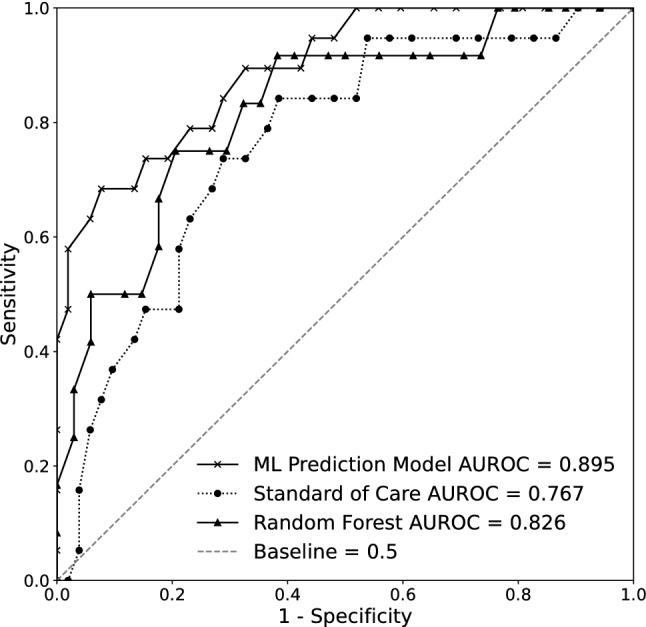
AUROCs demonstrating the superior performance of the machine learning prediction model by comparison with the standard of care comparator, and a random forest model. *AUROC* area under the receiver operator characteristic curve

The complete list of performance metrics for the ML prediction model, the random forest model, and the scores calculated for the standard of care comparator are shown in Table 4. The ROC curves for the hold-out test dataset of the ML prediction model, the hold-out test dataset of the random forest model, as well as for the standard of care comparator are shown in Fig. 4. The baseline curve in Fig. 4 represents a model that is equivalent to a random coin-flip, and unable to discriminate between the classes (i.e., types of ABA treatment plans). The ML prediction model achieved a strong performance for classifying patients as requiring comprehensive ABA treatment or requiring focused ABA treatment with an AUROC of 0.895 for the hold-out test dataset (95% CI 0.808–0.959). The random forest model achieved a good performance for classifying patients with an AUROC of 0.826 (95% CI 0.678–0.951). The ML prediction model significantly outperformed the standard of care comparator, which had an AUROC of 0.767 for the hold-out test dataset (95% CI 0.629–0.891). In addition, the ML prediction model also outperformed the random forest model. Both machine learning models, the ML prediction model and the random forest model outperformed the standard of care comparator. The ML prediction model is a binary classifier, and in this case, the “positive” class represents patients who have a ground truth of comprehensive ABA treatment, and the “negative” class represents patients who have a ground truth of focused ABA treatment. Calculations of sensitivity, specificity, positive predictive value, and negative predictive value were all greater for the ML prediction model compared with the standard of care comparator. At the chosen operating point, the ML prediction model outperformed the random forest model in sensitivity and negative predictive value and displayed similar positive predictive value. As all four metrics are related and specificity typically increases as sensitivity decreases, without a direct measure value of sensitivity of 0.789 for the random forest model, the direct comparison is not possible. However, as seen in Fig. 4, the random forest model sits at or below the ML prediction model specificity for nearly every sensitivity value. Both machine learning models sit at or above the standard of care specificity for nearly every sensitivity value.

**Table 4:** Performance metrics demonstrating the discriminative capabilities of the XGBoost-based machine learning prediction model by comparison with the random forest model and the standard of care comparator. Metrics used include AUROC, sensitivity, specificity, PPV, and NPV. The performance metrics are reported at operating points chosen to have the same sensitivity of 0.789 for both the ML prediction model and the standard of care comparator. All metrics include a 95% CI

Performance metrics	ML prediction model	Random forest model	Standard of care comparator
AUROC (95% CI)	0.895 (0.808–0.959)	0.826 (0.678–0.951)	0.767 (0.629–0.891)
Sensitivity (95% CI)	0.789 (0.673–0.906)	0.750 (0.615–0.885)	0.789 (0.700–0.878)
Specificity (95% CI)	0.808 (0.740–0.876)	0.824 (0.753–0.894)	0.635 (0.571–0.698)
PPV (95% CI)	0.600 (0.478–0.722)	0.600 (0.464–0.736)	0.441 (0.360–0.522)
NPV (95% CI)	0.913 (0.861–0.965)	0.903 (0.846–0.960)	0.892 (0.843–0.940)

*AUROC* area under the receiver operator characteristic curve; *CI* confidence interval; *PPV* positive predictive value; *NPV* negative predictive value

In addition to the ML prediction model, random forest model, and the standard of care comparator, the results of the 5 split k-fold cross-validation for the XGBoost-based model are consistent with the results of the final model. Across the 5 validation results, the mean AUROC was 0.877 and while an exact sensitivity of 0.789 was not measured, at the nearest measured operating point for each model, the mean sensitivity was 0.816 and the mean specificity was 0.803. These results suggest that the model did not overfit to the primary model’s holdout test set and the results are generalizable to a larger dataset.

**Fig. 5:**
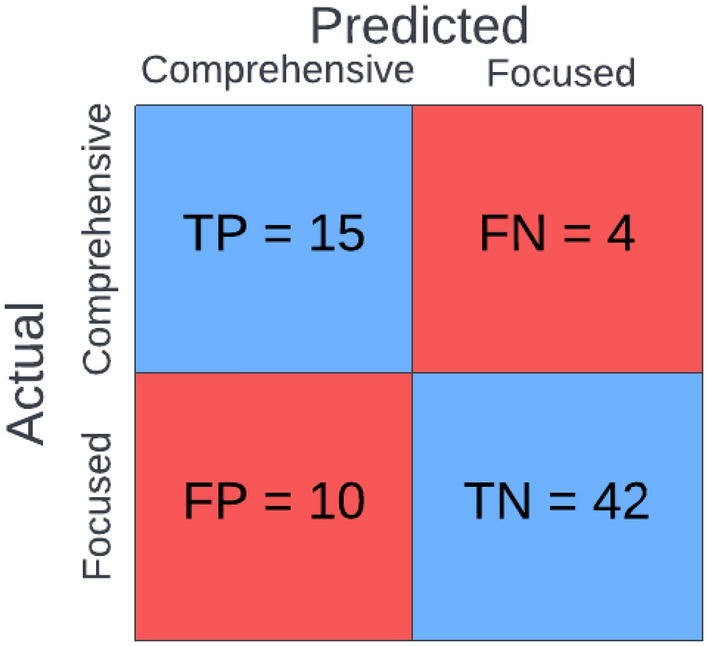
Confusion matrix providing a visual representation of the machine learning prediction model’s output for the hold-out test dataset. Out of the 71 patients in the hold-out test dataset, the ML prediction model successfully classified 57 patients as requiring comprehensive ABA treatment or focused ABA treatment. *ABA* applied behavioral analysis; *TP* true positive; *FP* false positive; *TN* true negative; *FN* false negative

At the chosen operating point for the ML prediction model (i.e., sensitivity: 0.789; specificity: 0.808), the prediction model was able to classify patients between the two ABA treatment plans with only 14 misclassifications out of the 71 total patients in the hold-out test dataset as shown in the confusion matrix in Fig. 5. It should be noted that the operating point for the ML prediction model was selected to prioritize true positives and limit false negatives, as will be discussed in more detail below (in the *Discussion* section). It should be further noted that the majority of misclassifications (false positives = 10 accounting for 71% of total misclassifications) indicate comprehensive ABA treatment for patients that may only require focused ABA treatment. A very small portion of the misclassifications (false negatives = 4 accounting for 28% of total misclassifications) indicated focused ABA treatment for patients that may require comprehensive ABA treatment.

### Feature importance

**Fig. 6:**
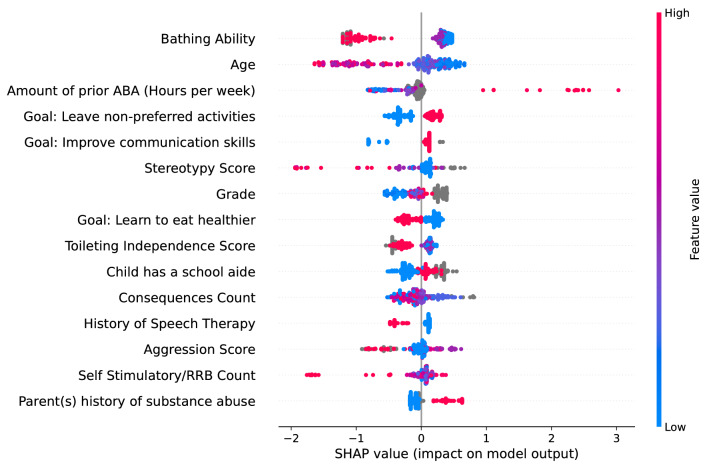
SHAP feature importance plot showing the 15 most important input features that contributed to the discriminative ability of the machine learning prediction model. *SHAP* SHapley Additive explanations; *ABA* applied behavioral analysis; *RRB* restrictive and repetitive behavior

A SHAP analysis was used to evaluate the importance of each input feature in generating the model’s output. It should be noted that the *Feature Selection based on SHAP values* described in the *Methods* section was used for feature pruning (along with other methods of feature selection) in order to obtain the feature matrix. The SHAP analysis as described here was applied to the prediction model to rank the individual contributions of features to the predictive ability of the model. This ranking was achieved by examining how each individual feature value that was used as a model input affected the classification of comprehensive versus focused ABA treatment while training the model (i.e., within the training dataset). Figure 6 shows the SHAP plot detailing the contributions of the 15 most important features in classifying patients as requiring either comprehensive ABA treatment plans (i.e., “positive” class) or focused ABA treatment plans (i.e., “negative” class). The SHAP plot ranks features by importance to model predictions top to bottom in the decreasing order of importance. Within the figure, the gray colored data points in the plot represent samples with null values for that particular input feature. The top three features which contributed most in the discriminative capabilities of the ML prediction model were bathing ability, age, and amount of past ABA treatment plans (hours per week). The features that contributed less substantially (i.e., the bottom three features of the plot) were aggression score, self-stimulatory/restricted, repetitive behaviors (RRB) count, and parent(s) history of substance abuse. However, as Fig. 6 showcases the top 15 most important features, those three features at the bottom of the SHAP plot were still in the middle range of overall importance within the 30 features used in the feature matrix. Various parent-expected goals, including goals to learn new ways to leave non-preferred activities and improving communication skills, were also among the top 10 most important features.

## Discussion

The aim of the present study was to determine whether a machine learning prediction model could classify (i.e., determine) the appropriate type of ABA treatment plan for patients diagnosed with ASD. We identified only one other study that used ML methods to predict ASD treatment recommendations, however, the focus of their study was predicting which treatment goals to target [34]. To the best of our knowledge, our study is the first to use ML to predict ABA treatment plan *type* using readily available information gathered solely from patient intake forms for patients referred to BCBAs for ABA treatment. Using a rigorous approach and feature selection process, the ML prediction model achieved excellent performance and revealed which feature inputs contributed most to the model’s predictions. For classifying patients as requiring comprehensive or focused ABA treatment, the ML prediction model achieved an AUROC of 0.895 in the hold-out test dataset, which exceeded the performance of the standard of care comparator and the performance of the random forest model, as shown in Fig. 4. Both machine learning models (i.e., XGBoost-based ML prediction model and random forest model) outperform the standard of care, indicating that machine learning more broadly offers substantial value in determining the appropriate type of treatment plan for an individual beginning ABA therapy. At the chosen operating point (i.e., sensitivity: 0.789; specificity: 0.808), all other performance metrics for the ML prediction model were also greater than those of the standard of care comparator. The ML prediction model was able to correctly classify ~ 80% of the ABA treatment plans in the hold-out test dataset, with the majority of misclassifications being false positives. Regarding input features, the SHAP analysis demonstrated that bathing ability, age, and amount of past ABA treatment plans had the greatest influence on the type of treatment plan prediction, providing some clinical insight into which features impact ABA treatment plan type determination. Collectively, our findings indicate that ML can be used as an aid in determining ABA treatment plan type for patients with ASD, providing a more standardized approach utilizing easily accessible information from patient intake forms.

It is widely accepted that ABA is the gold standard treatment for patients with ASD [8, 9, 10, 10, 11]. An early and appropriate ABA treatment plan is associated with an overall better quality of life, ranging from significant improvements in cognition, language, and adaptive behavior to more functional outcomes in later life [8, 2, 11]. Despite these findings, a major challenge in the treatment of patients with ASD is determining which ABA treatment plan will be most effective for a given individual patient. This is particularly true given the heterogeneity of this disorder, and the increasing number of newly trained BCBAs in the workforce to meet the demand of therapists qualified for delivering ABA treatment that must make individual determinations of treatment plan intensity for patients [13–18]. Further, the present standard of care for treatment plan type determination is intrinsically subjective and inconsistent [12]. The use of readily available patient information along with ML provides an opportunity to standardize ABA treatment plan type determination and aid BCBAs in determining the most appropriate plan for a given individual patient, ultimately leading to more effective treatment for more patients with ASD.

### ML prediction model feature selection

A key aspect of the present study was our rigorous feature selection process. Feature selection was crucial in reducing the dimensionality of the data and subsequently in selecting the inputs to achieve a strong performance, especially because of the small sample size of patients used in this study. Using a combination of various feature selection methods, 30 final features were selected from an initial set of 154 features. One of the key steps in the feature selection process was the combined AUROC-based feature elimination method. From the initially pruned down feature set of 83 features, and using the combined AUROC-based feature elimination method, we were able to further prune down the feature set to 30 features (Table 1) encompassing various aspects of the patients’ data. Often when there are more input features, the predictive task of the model is made more difficult, which is informally referred to as the “curse of dimensionality” [36]. The main objective of the feature selection process, in this case, was to reduce dimensionality in order to increase the model’s performance and to maintain model interpretability. The combined AUROC-based feature elimination method was successful in improving the model performance—the mean of cross-validation AUROC on the training dataset improved from 0.72 to 0.8, as shown in Fig. 2. Also shown in Fig. 2, there were a few points where the mean of cross-validation AUROC was ~ 0.8. The best performing feature sets were sets with between 12 and 30 features. The mean of cross-validation AUROC for the training dataset decreased as the dimensionality of the feature sets was reduced further, as illustrated by the tail on the right side of the plot in Fig. 2. From the candidate feature sets with 12 to 30 features, we chose the feature set that had the highest mean of cross-validation AUROC and also captured the most information across various facets of the patient data, which in this case was a set of 30 features. However, it should be noted that the AUROC performance difference between feature sets with 12 and 30 features does not appear significant in Fig. 2. Thus, in a hypothetical case of limited data availability, a model using a smaller number of inputs could be employed, without significant performance degradation. Generally, provided that a dataset would be large enough to support the evidence, it would be preferable to use a feature set with the least number of features.

### ML prediction model performance for ABA treatment plan prediction

Utilizing XGBoost, a gradient-boosted tree ensemble method of ML, we developed a prediction model utilizing readily available and easily accessible information from patient intake forms who were referred for ABA treatment. The ability of the ML prediction model to classify a comprehensive or focused ABA treatment plan was robust, achieving an AUROC of 0.895 (Fig. 4) with relatively high specificity and sensitivity (Table 4). At the selected operating points, the ML prediction model correctly classified ~ 80% of ABA treatment plans (Fig. 5). The operating points were selected to prioritize true positives and limit false negatives while maintaining a relatively high accuracy (accuracy is defined as the ratio of the number of correct classifications to the total number of classifications), and this is reflected in our findings that the majority (10 of 14) misclassifications in the hold-out test dataset were false positives. While a false positive result may recommend comprehensive ABA treatment to a patient who may benefit from just focused ABA treatment, a false negative may lead to insufficient treatment for a patient in need of comprehensive ABA treatment, which could reduce the benefits of ABA treatment for that particular patient. On the other hand, a false positive result could theoretically recommend more treatment than the patient may need, but this would not diminish the benefits of ABA treatment [54, 55]. In other words, a false positive can still provide a benefit to the patient, while a false negative might not. However, while it would be theoretically ideal to have all misclassifications be false positives, in practice, selecting an operating point that would further decrease the number of false positives leads to a significant decrease in accuracy by way of significantly increasing the overall number of misclassifications (i.e., via the addition of a significant number of false positives). Further, while misclassifications of false positives are preferred, increasing the number of false positives (e.g., in order to reduce false negatives) would have the undesired effect of detracting from treatment resources, for example by decreasing the availability of current professionals (e.g., BCBAs and RBTs).

To further examine the potential of machine learning for ABA treatment plan type determination and evaluate the best type of machine learning algorithm to deliver this prediction, the random forest model was developed as an additional comparator. The random forest model was able to identify the correct plan type with relatively high performance metrics, for example as indicated by an AUROC of 0.826. In addition, the random forest model outperformed the standard of care comparator. This indicates that machine learning can be a powerful tool in predicting the appropriate type of ABA treatment plan for an individual beginning ABA and shows potential to be a more capable tool than the currently utilized method. However, there were severe limitations to the random forest model compared to the XGBoost-based ML prediction model. First, the performance of the XGBoost-based ML prediction model is substantially higher than the performance of the random forest model, showcasing the benefits of the XGBoost algorithm over the random forest algorithm. Second, by comparison to the XGBoost-based ML prediction model, the random forest model requires additional data processing and filtering to enable its use. While the XGBoost-based ML prediction model can handle null values, the random forest model either needs to impute data or require a complete set of inputs for the model to run. Data imputation is a challenging approach, particularly with a condition as heterogeneous as ASD. Using extreme values to indicate null values can be utilized for certain types of data, but is not always an approach which can be generalized to all data types. The random forest model required filtering out some data points without all data present. While these methods for mitigating null values as described for the random forest model work in order to build a theoretical model for comparison purposes, in a real world scenario, it is very likely that data provided by the parents of patients with ASD may be incomplete. While filtering was performed to eliminate features with high levels of missingness, the overwhelming majority of patients (89%) in the total dataset had at least one missing feature. Without a robust imputation process or the ability to handle null values, many of these patients may not be eligible for a prediction from a machine learning model that is not developed with the XGBoost algorithm. This lack of information may hinder BCBAs in traditional determination methods as well, so the ability of the ML prediction model to work with null values adds additional advantages.

Given that the present standard of care for ABA treatment plan type determination is multifactorial and highly subjective and inconsistent [12], there is no comparator by which to compare the performance of the ML prediction model. Accordingly, we developed a standard of care comparator using features that are specified by the BACB in their treatment guidelines to contribute to the decision of a focused vs. a comprehensive ABA care plan [8, 11]. While this comparator achieved an AUROC of 0.767, the overall performance of the ML prediction model was superior to this comparator for every performance metric calculated (see Fig. 4,Table 3). It should be noted that the calculations and data analysis performed by the ML prediction model are far too complex to be performed manually by any individual. ML models are able to learn non-linear relationships between the input features and the outcome and also identify the relationships between the outcome and the interactions of multiple features.

To the best of our knowledge, this is the first study to develop and validate an ML prediction model utilizing data solely from patient intake forms with the goal of determining the ABA treatment plan type. However, Kohli et al. recently conducted a pilot study in which two ML models, Patient Similarity and Collaborative Filtering, were developed and tested to identify personalized treatment goals via domains and specific behavior targets for patients with ASD [34]. Both of their methods provide recommendations based on similarity between patients. The Patient Similarity method determines similarity by calculating the cosine similarity between patient vectors consisting of demographic information and assessment data to recommend treatment plans based on the treatment plans received by similar patients. The Collaborative Filtering method uses the patient demographics and assessment data to create profiles and recommends treatment plans based on patients with similar profiles. Their AUROC values for target recommendations were high (range: 0.78 and 0.80), however, their values for domain recommendations did not perform as well (range: 0.65 and 0.74). Some potential limitations of their study were the use of a relatively small number of patients (*n* = 29) and feature inputs that required in-depth assessment and score calculation by ASD-trained BCBAs or therapists, the latter which may limit the ability of the prediction models to generate immediate predictions in clinical settings. In contrast, our prediction model used inputs taken solely from patient intake forms and can be deployed to make immediate predictions for ABA therapy plan type using readily available patient information.

### ML prediction model feature importance

To gain insight into which features were important contributors in the classification of the ML prediction model, a SHAP analysis was employed and a few key findings that are of clinical interest should be noted. As shown in the SHAP summary plot (Fig. 6), a greater bathing ability and an older age were strongly associated with a focused ABA treatment plan type classification. This finding prompted us to further investigate the performance of the model for three subsets of the hold-out test dataset based on age (i.e., < 5 years old, 5 to < 8 years old, >  = 8 years old) in order to explore the effect of age, which is one of the most important factors when determining the treatment plan for any individual patient [54, 55, 12, 56]. The performance of the ML prediction model was superior to the standard of care comparator for every performance metric calculated in each age subgroup (see Additional file 1: Table S2 and Figures S1 and S2A-2C).

The third feature (in the order of decreasing importance in the SHAP summary plot displayed in Fig. 6), past ABA treatment (hours per week), indicates that those patients who had greater levels of past ABA treatment were more likely to receive comprehensive ABA treatment. Other features on the list of the 10 most important features include various goals set for the patients. The type of goals set for the patient are usually used by BCBAs to determine the type and duration of the ABA treatment [8, 11]. Additionally, inputs about the level of various day to day skills and behaviors of the patients such as toileting, aggression and self stimulatory/restricted and repetitive behaviors, which play a key role in determining the treatment plan type, were also among the most important features.

### Experimental limitations

While the use of ML for ABA treatment plan type determination is innovative and novel, there are a few noteworthy experimental limitations. First, compared with studies employing ML and relatively larger data sets [57, 58], the present study included a relatively smaller sample size. This is particularly true for the hold-out test dataset (*n* = 71), as this comprised 20% of the total sample size. Second, in the present study, there was a larger number of patients (~ 72%) in the focused ABA treatment group (ground truth) than the comprehensive ABA treatment group (ground truth). In the present study, the ratio between patients in the focused ABA treatment group (ground truth) and the patients in the comprehensive ABA treatment group (ground truth) was ~ 2.6. In a policy report with a larger population size (i.e., 1879 patients), it was indicated that more patients underwent focused ABA treatment versus comprehensive ABA treatment, however, their ratio between patients in the focused ABA treatment group and the patients in the comprehensive ABA treatment group was only ~ 1.3 [59]. As such, future studies should include a larger sample size and a relatively more balanced number of patients in comprehensive and focused ABA treatment groups. Further, future prospective studies will be needed to determine whether the use of ML algorithms for ABA treatment recommendations leads to more favorable outcomes in patients with ASD. Finally, it should be noted that the ground truth data for the 359 patients included in this retrospective study do not reflect a consensus of BCBAs, but rather individual BCBA determinations from several different BCBAs. While inconsistencies and a lack of standardized approach for any given BCBA may be reflected in our full dataset, having ABA treatment plan type determinations (i.e., ground truth determinations) from several different BCBAs helps mitigate some inconsistencies (as opposed to one BCBA being responsible for all 359 ground truth determinations). Future studies could include BCBA consensus on ground truth determinations, and/or a larger number of BCBAs to further mitigate inconsistencies and a lack of standardized approach for any given BCBA.

## Conclusions

ASD is a complex, life-long neurodevelopmental disorder for which the measured prevalence continues to increase. ABA treatment has long been recognized as the gold standard treatment for ASD, however the present standard of care for ABA treatment plan type determination is highly subjective and inconsistent. The findings from the present study demonstrate that ML can be used to classify the appropriate ABA treatment plan type from readily available information derived from patient intake forms for patients who have been diagnosed with ASD and referred to BCBAs for ABA treatment. Starting with patient intake forms containing a wealth of data, we employed a rigorous feature selection process and identified the best performing feature sets as having between 12 and 30 features. While a set with 30 features provides for capturing most information across various aspects of the patient data, feature sets with a lower number of features (e.g., 12 features) could be employed (for example when limited data availability is a drawback), without significant model performance degradation. The robust ability of our ML prediction model to accurately classify could help standardize the determination of the appropriate ABA treatment plan type, and aid BCBAs in this process. This could ultimately lead to more effective ABA treatment for more patients with ASD.

## Supplementary Information

**Additional file1: Table S1.** List of all inputs from various categories available after processing the data obtained from the applied behavioral analysis (ABA) patient intake forms. The table also indicates in bold font certain features that were derived from a combination of input features obtained from the ABA patient intake forms. **Table S2**. Performance metrics demonstrating the discriminative capabilities of the machine learning prediction algorithm (MLPA) by comparison with the standard of care (SOC) comparator in three different age groups (i.e., < 5 years, 5 to < 8 years, >= 8 years). Metrics used include area under the receiver-operator characteristic curve (AUROC), sensitivity, specificity, positive predictive value (PPV), and negative predictive value (NPV) for the three age groups, demonstrating the superior performance of the MLPA in all three age groups. All metrics include a 95% confidence interval (CI). Abbreviations: machine learning prediction algorithm (MLPA); standard of care (SOC); area under the receiver-operator characteristic curve (AUROC); confidence interval (CI); positive predictive value (PPV); negative predictive value (NPV). **Figure S1.** Confusion matrices providing a visual representation of the machine learning prediction algorithm’s (MLPA’s) output for the hold-out test dataset in three different age groups (i.e., < 5 years, 5 to < 8 years, >= 8 years). Abbreviations: comprehensive (Comp.), true positive (TP); false positive (FP); true negative (TN); false negative (FN). **Figure S2.** Area under the receiver-operator characteristic curves (AUROCs) demonstrating the superior performance of the machine learning prediction algorithm (MLPA) by comparison with the standard of care comparator in three different age groups (i.e., < 5 years, 5 to < 8 years, >= 8 years). The baseline curve represents a model that is equivalent to a random coin-flip, and unable to discriminate between the classes (i.e., types of applied behavioral analysis (ABA) treatment plans). Abbreviations: machine learning prediction algorithm (MLPA); area under the receiver-operator characteristic curve (AUROC).

## Data Availability

The datasets generated and/or analyzed during the current study are not publicly available as data are proprietary.

## Abbreviations

ASD: Autism spectrum disorder
ABA: Applied behavioral analysis
ML: Machine learning
AUROC: Area under the receiver-operating characteristic curve
PPV: Positive predictive value
NPV: Negative predictive value
CDC: Centers for Disease Control and Prevention
US: United States
BCBA: Board Certified Behavior Analyst
DSM-5: Diagnostic and Statistical Manual of Mental Disorders, Fifth Edition
RBT: Registered behavior technician
EHRs: Electronic health records
SHAP: SHapely Additive exPlanations
BACB: Behavior Analyst Certification Board
CI: Confidence interval
ADHD: Attention-deficit hyperactivity disorder
