# Supplementary material for: Machine learning determination of applied behavioral analysis treatment plan type
Source: Brain Inform. 2023 Mar 2;10(1):7. doi: 10.1186/s40708-023-00186-8 (PMC9981822; doi:10.1186/s40708-023-00186-8)
Supplement: Supplementary file 1 — Additional file1: Table S1. List of all inputs from various categories available after processing the data obtained from the applied behavioral analysis (ABA) patient intake forms. The table also indicates in bold font certain features that were derived from a combination of input features obtained from the ABA patient intake forms. Table S2. Performance metrics demonstrating the discriminative capabilities of the machine learning prediction algorithm (MLPA) by comparison with the standard of care (SOC) comparator in three different age groups (i.e., < 5 years, 5 to < 8 years, >= 8 years). Metrics used include area under the receiver-operator characteristic curve (AUROC), sensitivity, specificity, positive predictive value (PPV), and negative predictive value (NPV) for the three age groups, demonstrating the superior performance of the MLPA in all three age groups. All metrics include a 95% confidence interval (CI). Abbreviations: machine learning prediction algorithm (MLPA); standard of care (SOC); area under the receiver-operator characteristic curve (AUROC); confidence interval (CI); positive predictive value (PPV); negative predictive value (NPV). Figure S1. Confusion matrices providing a visual representation of the machine learning prediction algorithm’s (MLPA’s) output for the hold-out test dataset in three different age groups (i.e., < 5 years, 5 to < 8 years, >= 8 years). Abbreviations: comprehensive (Comp.), true positive (TP); false positive (FP); true negative (TN); false negative (FN). Figure S2. Area under the receiver-operator characteristic curves (AUROCs) demonstrating the superior performance of the machine learning prediction algorithm (MLPA) by comparison with the standard of care comparator in three different age groups (i.e., < 5 years, 5 to < 8 years, >= 8 years). The baseline curve represents a model that is equivalent to a random coin-flip, and unable to discriminate between the classes (i.e., types of applied behavioral analysis (ABA) treat [file 40708_2023_186_MOESM1_ESM.docx]

**Additional files**

**Table S1:** List of all inputs from various categories available after processing the data obtained from the applied behavioral analysis (ABA) patient intake forms. The table also indicates in bold font certain features that were derived from a combination of input features obtained from the ABA patient intake forms.

| **Input Categories** | **Input Features** |
| --- | --- |
| Demographics | Age |
|  | Sex |
|  | Is the patient multilingual? |
|  | Is English the primary language? |
|  | Child’s parents have a divorce decree |
| Schooling | Attends School? |
|  | Grade (If attends school) |
|  | Is the child in a general education classroom? |
|  | Child has individualized education plan (IEP) or admission, review, and dismissal (ARD) |
|  | Child received additional services as part of IEP/ARD |
|  | Child has a school aide/support during school hours |
| Parent’s Medical History | Mother or Father has history/presence of depression or manic-depression |
|  | Mother or Father has history/presence of autism spectrum disorder (ASD) |
|  | Mother or Father has history/presence of substance abuse or dependence |
|  | Mother or Father has history/presence of anxiety disorders (OCD, phobias, etc.) |
|  | Mother or Father has history/presence psychosis/schizophrenia |
|  | Mother or Father has history/presence of learning disability |
|  | Mother or Father has history/presence of attention deficit hyperactivity disorder (ADHD) |
| Seizure | Child has had seizures in the past? |
|  | Is a seizure plan in place for the child? |
| Treatment/Therapy | Child has current ABA treatment |
|  | Child has had any ABA treatment(current or past) |
|  | Child has received prior ABA treatment |
|  | Amount of prior ABA treatment(hours of treatment per week) |
|  | Amount of prior ABA treatment(years) |
|  | History of Physical Therapy: Has the patient ever received Physical Therapy? |
|  | History of Occupational Therapy: Has the patient ever received Occupational Therapy? |
|  | History of Speech Therapy: Has the patient ever received Speech Therapy? |
|  | History of Other Therapy: Has the patient ever received any other Therapy? |
| Behavioral and Skill Related Inputs | Behavioral Assessment |
|  | Does the child display aggression? |
|  | How frequently does the child exhibit aggression? |
|  | How severe is the child's aggressive behavior? |
|  | Does the child engage in self-injury? |
|  | How frequently does the child engage in self-injury? |
|  | Does the child engage in stereotypy? |
|  | How frequently does the child exhibit stereotypy? |
|  | Does the child destroy property? |
|  | How frequently does the child destroy property? |
|  | **Aggression Score: Level of the patient’s engagement in aggressive behavior derived from the frequency and severity of aggression** |
|  | **Stereotypy Score: Level of the patient’s engagement in Stereotypical repetitive behavior derived from the frequency and severity of stereotypy behavior** |
|  | **Self Injury Score: Level of the patient’s engagement in self injury derived from the frequency and severity of the patient’s engagement in self injury** |
|  | **Destroy Property Score: Level of the patient’s engagement in destroying property derived from the frequency and severity of the patient’s engagement in destroying property** |
|  | Child is described as easy-going or "go with the flow" |
|  | Child is described as anxious or easily upset by abnormal things |
|  | Does the child usually follow simple directions in the home setting? |
| Consequences for Misbehavior | Consequence for misbehavior is to ignore behavior |
|  | Consequence for misbehavior is to verbally reprimand child |
|  | Consequence for misbehavior is to redirect child to another activity |
|  | Consequence for misbehavior is to negotiate/reason with child |
|  | Consequence for misbehavior is to threaten to remove privileges |
|  | Consequence for misbehavior is to actually remove privileges |
|  | Consequence for misbehavior is to spank child |
|  | Consequence for misbehavior is to give child a time-out |
|  | Consequence for misbehavior is to send child to room |
|  | **Consequences Count - How many of the 'consequences for misbehavior' options were checked off** |
| Communication Skills | Communication Ability - child communicates vocally (with words/sentences, asks questions, etc.) |
|  | Communication Ability - child communications through gestures and physical touch |
|  | Communication Ability - child communicates with devices or through pictures |
|  | Primary Communication Method - verbal |
|  | Primary Communication Method - gestures |
|  | Primary Communication Method - behaviors |
|  | Primary Communication Method - grabbing |
|  | Primary Communication Method - pictures |
|  | Primary Communication Method - devices |
|  | Primary Communication Method - pointing |
|  | Primary Communication Method - guiding |
|  | Primary Communication Method - sign language |
|  | Child had speech abilities which were lost |
|  | Was there illness at the time of the child losing the ability to speak? |
|  | Understanding - parent can usually understand child |
|  | Understanding - strangers can usually understand child |
|  | Understanding - child can hold a conversation about their favorite topic |
|  | Listening - Child can follow vocal instructions without cues |
|  | Listening - Level of child's understanding of parents |
| Feeding and Drinking Habits | Child can feed themselves with a spoon or fork (independently) |
|  | Child can feed themselves finger foods (independently) |
|  | Child needs some or significant help eating with utensils |
|  | Child needs assistance for both finger feeding and using utensils |
|  | Child eats most foods |
|  | Child is a picky eater |
|  | Child refuses some foods |
|  | Child loves some foods |
|  | Child has food allergies |
|  | Child has food sensitivities |
|  | Child requires a special diet |
|  | Child has issues with certain food textures |
|  | Child's family eats together |
|  | Child is currently or has previously seen a feeding specialist |
|  | Child can drink from a glass |
|  | Child can drink from a sippy cup |
|  | Child can drink from a bottle |
|  | Child can drink with a straw |
|  | **Food Choice Score: Score indicating food choice behaviors** |
|  | **Drinking Ability Score: Score indicating drinking skills** |
| Toileting and Bathing Skills | Child can use the toilet independently |
|  | Child requires parent assistance for toileting |
|  | Child can use the toilet during the daytime |
|  | Child has some toilet accidents |
|  | Child is solely bladder trained |
|  | Child wears a diaper or pull-up at night |
|  | Child has difficulty with constipation |
|  | Child has difficulty with diarrhea |
|  | Child is not potty trained |
|  | Child's ability to dress themselves |
|  | Child's ability to undress themselves |
|  | Child prefers bathing |
|  | Child prefers showers |
|  | Bathing Ability - Ability of patients to bathe themselves |
|  | **Toileting Independence Score: Score indicating independence in toileting skills** |
| Stim/RRB | Stim/RRB - engages in hand-flapping |
|  | Stim/RRB - engages in toe-walking |
|  | Stim/RRB - rocks torso/upper body |
|  | Stim/RRB - lines up toys |
|  | Stim/RRB - looks at things out of corner of eye or indirectly |
|  | Stim/RRB - smells odd items one would not typically smell |
|  | Stim/RRB - eats or places inedible items in mouth |
|  | Stim/RRB - repeats the same lines from movies or videos |
|  | Stim/RRB - insist on things being done the same way every time |
|  | Stim/RRB - tries to direct actions of parents or others in home |
|  | Stim/RRB - gets upset at interruptions or changes in routine |
|  | Stim/RRB - is unusually attached to toys or objects |
|  | Stim/RRB - insists on carrying objects around |
|  | Stim/RRB - difficulty leaving objects at home |
|  | Stim/RRB - engages in repetitive activities which may be odd |
|  | **Stim/RRB Count: Count of Stim/RRB options checked for a patient** |
| Social Skills | Social Skills - child has friends |
|  | Social Skills - child wants to have friends |
|  | Social Skills - tends to gravitate towards older children |
|  | Social Skills - prefers playing alone |
|  | Social Skills - has difficulty playing with other children |
|  | Social Skills - has age appropriate toy play skills |
|  | Social Skills - exhibits odd behaviors when with other children |
|  | Social Skills - plays any group sports |
|  | Social Skills - belongs to any clubs or organizations |
|  | Social Skills - has been removed from a social setting due to behavior |
|  | Social Skills - shows affection to parent and/or other family |
|  | Social Skills - notices when parent is upset or hurt |
|  | Social Skills - makes eye contact with others |
|  | Social Skills - looks at parent when his/her name is called |
|  | Social Skills - utilizes eyes to gain parents' attention |
|  | Social Skills - responds to his/her name being called |
|  | Social Skills - seeks comfort from parent when upset |
|  | Social Skills - greets parent or other family members when they enter house/room |
|  | Social Skills - interested in activities of other family members |
|  | Social Skills - attempts to join in activities or plays of others |
|  | Social Skills - asks parents to join his/her play |
|  | Social Skills - responds better to some people vs. others |
|  | **Social Count: Score indicating social skills** |
| Sleep and Wake Patterns: | Sleep and Wake Patterns - Is a good sleeper? |
|  | Sleep and Wake Patterns - Wake up at night? |
|  | Sleep and Wake Patterns - Sleeps through the night? |
|  | Sleep and Wake Patterns - Appears rested in the morning? |
|  | Sleep and Wake Patterns - Has a bedtime routine? |
|  | Sleep and Wake Patterns - Has a regular bedtime? |
|  | Sleep and Wake Patterns - Wakes up at the same time? |
|  | Sleep and Wake Patterns - Takes medication for sleep? |
| Expected Parent Goals | Expected Parent Goals - improve communication skills |
|  | Expected Parent Goals - improve social skills |
|  | Expected Parent Goals - improve ability to participate in family activities |
|  | Expected Parent Goals - decrease challenging behaviors |
|  | Expected Parent Goals - get along better with parents and/or siblings |
|  | Expected Parent Goals - learn toilet training |
|  | Expected Parent Goals - learn to eat healthier/more balanced diet |
|  | Expected Parent Goals - learn to be more independent |
|  | Expected Parent Goals - new ways to express frustration or when upset |
|  | Expected Parent Goals - new ways to leave non-preferred activities |
|  | Expected Parent Goals - do what he/she is told without responding inappropriately |
|  | Expected Parent Goals - keep his/her body and others around him/her safe |
|  | Expected Parent Goals - increase participation in general education classroom/settings |
|  | Expected Parent Goals - increase flexibility and be less rigid |
| Medical History | ADHD Diagnosis |
|  | Anxiety Diagnosis |
|  | Depression Diagnosis |
|  | Communication and Speech related Diagnosis |
|  | Diagnoses of other medical conditions |
|  | Medication for ASD, ADHD, Anxiety, Depression, Behavior or Mood related conditions |
|  | Medication for Sleep |
|  | Medication for Allergies |
|  | Medication for other medical conditions |

**Table S2**: Performance metrics demonstrating the discriminative capabilities of the machine learning prediction algorithm (MLPA) by comparison with the standard of care (SOC) comparator in three different age groups (i.e., < 5 years, 5 to < 8 years, >= 8 years). Metrics used include area under the receiver operator characteristic curve (AUROC), sensitivity, specificity, positive predictive value (PPV), and negative predictive value (NPV) for the three age groups, demonstrating the superior performance of the MLPA in all three age groups. All metrics include a 95% confidence interval (CI). Abbreviations: machine learning prediction algorithm (MLPA); standard of care (SOC); area under the receiver operator characteristic curve (AUROC); confidence interval (CI); positive predictive value (PPV); negative predictive value (NPV).

| **Performance Metrics** | **Age < 5 years** | | **Age 5 to < 8 years** | | **Age >= 8 years** | |
| --- | --- | --- | --- | --- | --- | --- |
| **Number of Patients** | 25 | | 19 | | 27 | |
| **Model** | MLPA | SOC | MLPA | SOC | MLPA | SOC |
| **AUROC**  **(95% CI)** | 0.853  (0.698 - 0.971) | 0.711  (0.500 - 0.88) | 0.798  (0.550 - 1.00) | 0.596  (0.288 - 0.869) | 0.889  (0.615 - 1.00) | 0.833  (0.609 - 1.00) |
| **Sensitivity (95% CI)** | 0.857  (0.674 - 1.00) | 0.857  (0.674 - 1.00) | 0.500  (0.100 - 0.900) | 0.500  (0.100 - 0.900) | 0.667  (0.133 - 1.00) | 0.667  (0.133 - 1.00) |
| **Specificity (95% CI)** | 0.474  (0.249 - 0.698) | 0.316  (0.107 - 0.525) | 0.947  (0.847 - 1.00) | 0.632  (0.415 - 0.848) | 1.00  (1.00 - 1.00) | 0.833  (0.684 - 0.982) |
| **PPV**  **(95% CI)** | 0.545  (0.337 - 0.754) | 0.480  (0.284 - 0.676) | 0.750  (0.326 - 1.00) | 0.300  (0.016 - 0.584) | 1.00  (1.00 - 1.00) | 0.333  (0.044 - 0.711) |
| **NPV**  **(95% CI)** | 0.818  (0.590 - 1.00) | 0.750  (0.450 - 1.00) | 0.857  (0.707 - 1.00) | 0.800  (0.598 - 1.00) | 0.96  (0.883 - 1.00) | 0.952  (0.861 - 1.00) |

**Figure S1:** Confusion matrices providing a visual representation of the machine learning prediction algorithm’s (MLPA’s) output for the hold-out test dataset in three different age groups (i.e., < 5 years, 5 to < 8 years, >= 8 years). Abbreviations: comprehensive (Comp.), true positive (TP); false positive (FP); true negative (TN); false negative (FN).

**
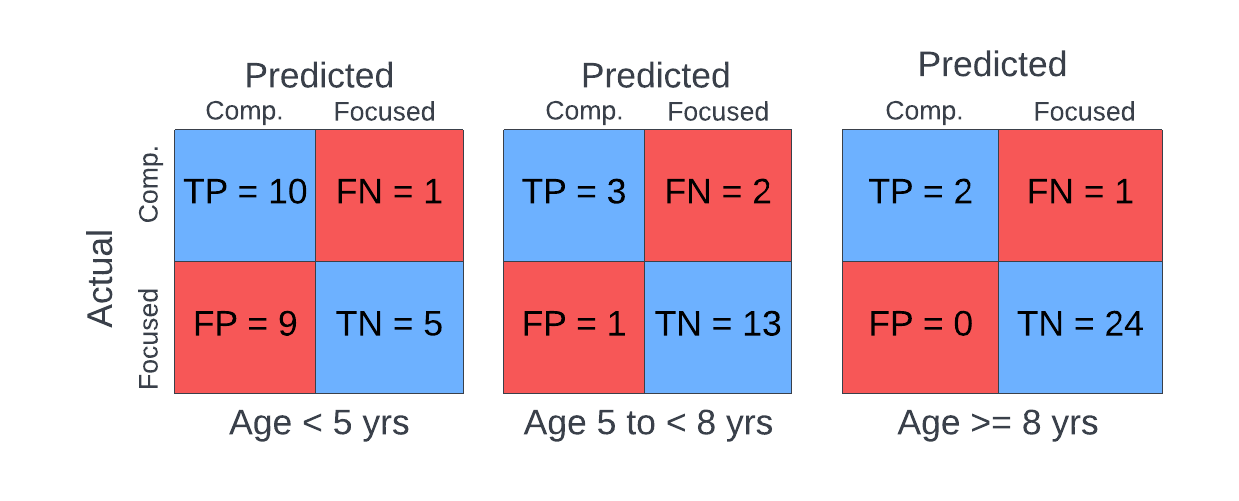
**

**Figure S2:** Area under the receiver operator characteristic curves (AUROCs) demonstrating the superior performance of the machine learning prediction algorithm (MLPA) by comparison with the standard of care comparator in three different age groups (i.e., < 5 years, 5 to < 8 years, >= 8 years). The baseline curve represents a model that is equivalent to a random coin-flip, and unable to discriminate between the classes (i.e., types of applied behavioral analysis (ABA) treatment plans). Abbreviations: machine learning prediction algorithm (MLPA); area under the receiver operator characteristic curve (AUROC).

**2A. Age Group: < 5 years old**

**
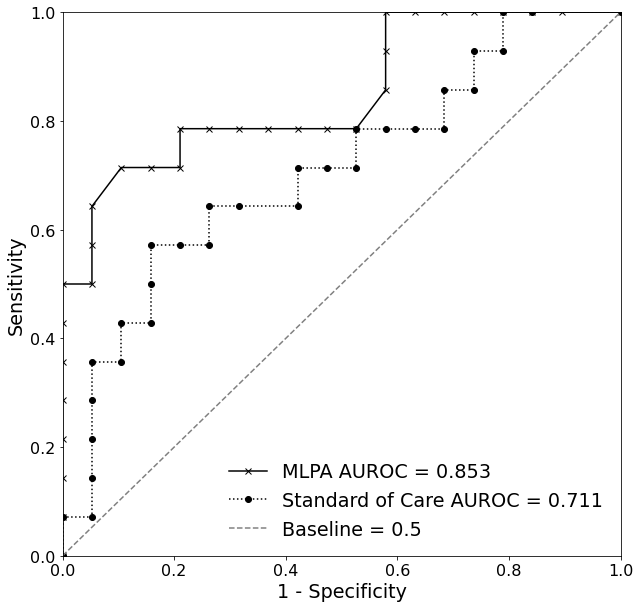
**

**2B. Age Group: 5 to < 8 years old**

**
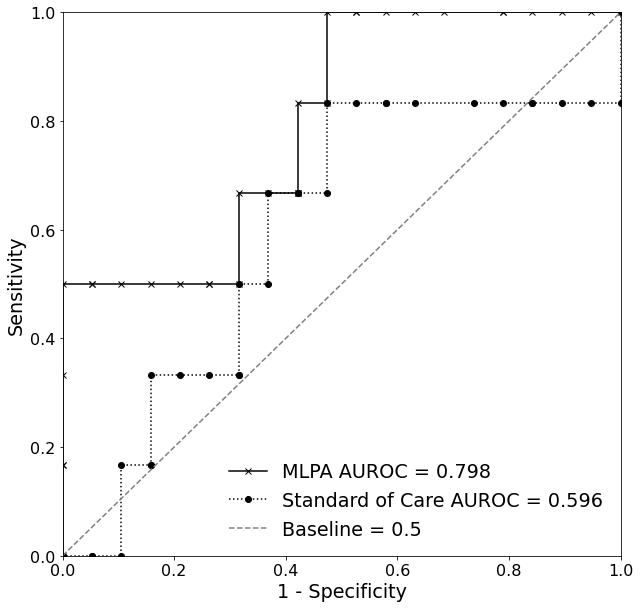
**

**2C. Age Group: >= 8 years old**

**
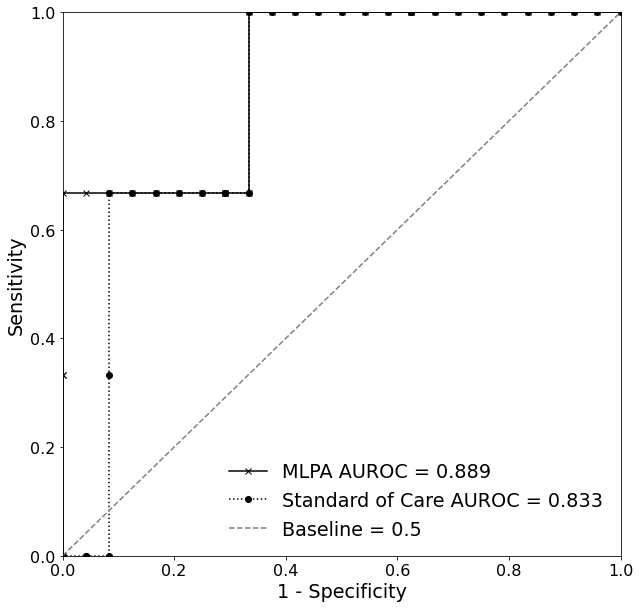
**
